# Medical liability claims in gynaecologic care: retrospective analysis of claims related to gynaecology in the Netherlands (2005–2022) – Is there a connection between treatment indication, phase of treatment and the risk of medical malpractice claims?

**DOI:** 10.1186/s12913-024-11943-8

**Published:** 2024-11-25

**Authors:** Désirée Klemann, Rankie ten Hoopen, Helen Mertens, Frits van Merode

**Affiliations:** 1 Department of Gynaecology and Obstetrics, Maastricht University Medical Centre+, Maastricht, 6229 HX the Netherlands; 2 Care and Public Health Research Institute, Maastricht University Medical Centre+, Maastricht, 6229 HX The Netherlands; 3 Assistant professor Faculty of Law, Maastricht University, Maastricht, 6211 LH the Netherlands; 4 Scientific Employee Boels Zanders Advocaten, 6199 AG Maastricht-Airport, the Netherlands; 5 Executive Board, Maastricht University Medical Centre+, Maastricht University, Maastricht, 6229 HX The Netherlands; 6 Maastricht University Medical Centre+, Maastricht, 6229 HX The Netherlands

**Keywords:** Gynaecology and obstetrics, Malpractice claims, Litigation, Indemnity payment, Treatment indications, Treatment phase, Surgical treatment, Incident, Informed consent, File management

## Abstract

**Background:**

An increased interest in medical liability claims has been noticed. Nevertheless, detailed data on subject of claims and possible factors that contribute to litigation and indemnity payments are scarce and relatively dated. Insight into these data may provide valuable information to prevent both incidents and malpractice claims.

**Objective:**

To analyse the subject, outcome and costs of malpractice claims related to gynaecological care and their connection with treatment indications and treatment phases.

**Design:**

A retrospective analysis of malpractice claims related to gynaecology.

**Setting:**

All claims related to gynaecology, filed and closed by Netherlands’ largest liability insurance company, Centramed between 2005 and 2022.

**Sample:**

*N* = 382.

**Methods:**

An in-depth analysis of claim files was performed.

**Results:**

A total of 68.6% of the claims were related to perioperative incidents. A total of 88.0% of all claims were related to treatments with a benign indication and only 12.0% were related to malignancies. The share of malignant treatment indications was high for claims related to diagnostic incidents (37.9%), compared to 7.3% for claims related to surgical treatment. Liability was accepted in 22.5% of all claims. The total costs of all claims amount €6,6mlj. Besides the indication for treatment, deficient expectation management (a lack of informed consent) contributes to dissatisfaction and increases the risk of malpractice claims. Finally, an inadequate medical file compromises legal defence and influences the judgement and settlement of malpractice claims.

**Conclusions:**

There is a connection between treatment indications and treatment phases and the risk of malpractice claims and their outcome.

## Background

Medical incidents and malpractice claims have a great impact on patients (and their relatives), healthcare professionals, healthcare organizations and liability insurance companies. Healthcare workers, hospital board members and policy makers are increasingly interested in trends and developments in malpractice claims not only because of the costs, but mainly from a quality-of-care perspective. An increase in the number of malpractice claims and the associated costs was first noticed in the U.S.A [1]. Recent European data from the United Kingdom and the Netherlands show a more or less stable number of submitted medical liability claims against hospitals, but a remarkable increase in costs (litigation costs, legal costs and damage compensation to patients and their relatives) [2, 3]. This increase in costs is mainly caused by relatively few claims with excessively high damage burdens, while in most claims, the costs and damages paid remain stable [3].

For the last decades, both European and American studies have concluded that general- and trauma surgeons, orthopaedists and gynaecologists are the top three medical specialties with the greatest number of claims filed and the highest damage burden [2, 4, 5]. Analysis of the subject and number of claims and their costs per medical specialty indicate that most claims are related to surgical specialties rather than to non-surgical and supporting specialties. Previous research showed that the top three specialties and the ratio between surgical and non-surgical specialties cannot be explained by the financial turnover (as a proxy for the production numbers) per medical specialty [3].

Gynaecology is one of the top three medical specialties with the most and highest liability claims [3, 4]. This medical specialty has both surgical as non-surgical characteristics. The subjects, characteristics and findings of gynaecological claims may therefore reflect multiple other medical specialties. Although each specialty has its own nature and characteristics, an in-depth analysis of gynaecological claims may contribute to more insight into the differences between surgical and non-surgical activities and related malpractice claims.

Detailed data on the subject of claims and possible factors that contribute to litigation and indemnity payments are scarce and relatively dated. It is understandable that when severe patient disability occurs (whether or not due to negligence), people are more willing to file a claim. The findings of Gómez et al. [5] and Chauhan et al. [6] confirmed this; hysterectomy-related problems, injury to the gastrointestinal system and missed diagnosis of cancer were the most frequently claimed events. Unfortunately, the studied subjects of claims remain categorized, general and often vague.

The final judgements of claims should be primarily related to the healthcare providers’ actions (deviating from the standard of care). Nevertheless, American studies suggest that in certain cases the judgements appear (more) connected to the severity of the patients’ health injury. Brennan [7] states that the key predictor of *acceptance* of liability and indemnity pay-out to the patient is not the presence of provider negligence, but the degree of patient disability. In rare cases, serious injuries led to settlements even without the occurrence of negligence. This would mean that a doctor could be held liable for an unexpected negative outcome, without negligence. However, these findings are based on a very small sample size (*N* = 51), are relatively dated (1996) and are highly dependent on the applicable legal system. Tom Baker on the other hand resoundingly disposes the so-called *myth* that juries award damages even when doctors have not been negligent [8]. Once liability is recognized, the severity of the patient’s injury does determine the extent of liability and financial compensation. We can imagine that in cases of significant health injury or an obvious medical mistake or negligence, patients may more easily find a (legal) representative, possibly leading to a stronger litigation process and (substantial) more compensation.

Research into the subject of (acknowledged) malpractice claims might provide valuable insight into quality of care from a patient perspective. However, this might also contribute to insight into the difference between surgical and nonsurgical specialties, and the specialty-related risk of malpractice claims. Therefore, we performed a detailed, qualitative in-depth analysis of all malpractice claims related to gynaecological cases, submitted to medical liability insurance company Centramed between 2005 and 2022 (*N* = 382). Our primary goal is to analyse the subject of claims and to find out whether claims related to a surgical specialism mostly concern the surgical process. We also aimed to determine in which treatment phase incidents occur that lead to a claim, and which factors related to claims lead to acknowledgement of liability and indemnity payments. We also wanted to test the hypothesis that the indication for treatment (referring to the nature of the underlying medical condition; benign versus malignant) influences the likelihood of liability claims.

## Methods

### Data collection

For our analysis, we searched the database of Centramed. Centramed is the largest medical liability insurance company of the Netherlands. Centramed currently provides liability insurance for over 50% of all Dutch hospitals.

### Inclusion and exclusion criteria

All claims filed and closed between 2005 and 2022, containing the search term ‘gynaecology’ as primary or secondary medical specialty involved, were included (reference date of data selection: 31.12.2022). A total of 437 claims met the inclusion criteria. Of these claims, 41 claims were still pending, while 396 claims were closed and available for an in-depth analysis, including the legal data, final judgement and financial data. After reading the summary of the claims, 14 claims were excluded from further analysis since they focused on incidents involving other medical specialties. The remaining 382 claims were included and analysed.

The data were collected based on anonymised information from the insurance company. All information was collected and reviewed by the first author, who is both a gynaecologist and a jurist, specialized in medical liability. Review took place at the insurance company office and the data mentioned below were anonymously collected, scored and stored.

### Data extraction

Table 1 describes the data that were extracted from the medical claim files. A claim may include multiple claim-elements (such as a surgical error and a delay in recognizing the complication and lack of informed consent).

**Table 1:** Data and information that was extracted from the medical claim files

Procedural data	Date of incident	
	Data of claim made	
	Data of claim closed	
Patient characteristics	Patient’s gender	Male/ female/ unknown
	Date of birth	
	Patient’s age	
Medical information	Diagnosis	
	Indication for treatment	Benign/malignant
	Description/summary of the incident	
Subject of claim	Phase of treatment	- Diagnostic phase - Non-surgical treatment phase - Surgical phase ◦ Pre-operative ◦ Perioperative ◦ Postoperative ◦ Per- and postoperative
	Other medical (sub)specialism involved	If yes, which
	Claim element	- Wrong/delayed diagnoses - Wrong/delayed treatment indication - Lack of knowledge - Medication error - Surgical error - Therapeutic failure - Delay in recognizing a complication - Lack of follow-up - Informed consent related - Inadequate medical file management - Organizational errors - Others
Legal data	Outcome of claim	- Accepted - Rejected - Settlement made
	Description/summary of legal handling	
Financial data	Total costs of claims	
	Pay-outs to patients/claimants	

### Dutch legal liability system and definitions

In the Netherlands patients who have suffered physical or material damage as a result from a (alleged) medical incident can file a medical liability claim to receive a financial compensation for their harm. Healthcare-related damage, does not automatically lead to liability of the healthcare provider. It is for the patient that files a claim (the plaintiff) to prove that all legal conditions for liability have been met. The legal conditions concern: (1) there must be damage (either physical or material), (2) there must be a fault or negligence from the healthcare provider, (3) there must be a causal connection between fault and healthcare damage and (4) the fault must be attributable to the healthcare provider.

In practice, a great majority (estimated 95%) of all medical liability claims are settled in an out-of-court setting. Normally, the patient addresses the claim to the healthcare provider involved. Healthcare providers are generally (collectively per hospital) insured for medical liability and will most likely refer the claim to their insurer, such as Centramed. The insurer will further handle the claim and assess whether the patient can prove that all legal conditions for liability have been met. Often, in the assessment of a liability claim, medical and legal experts’ advise(s) are used to conclude whether or not liability is acknowledged, rejected or a settlement is made. Only if all requirements are met, liability is *acknowledged* and all causal (health)injury (determined by injury experts) will be compensated. When the patient and the insurer fail to agree on the question of liability or the amount of compensation to be paid, the patient can submit the liability claim to a civil court. There are other procedures available for (dissatisfied) patients that were injured after healthcare. For example, a complaints procedure with the complaints officer of the hospital, a complaint by the Medical Disciplinary Board, or a complaint by a Disputes Committee. This committee can also award (relatively small) compensations, up to a maximum of €25,000. These procedures fall beyond the scope of this manuscript.

The liability insurance companies will *reject* a claim, even if an adverse event or incident occurred, if no medical negligence or malpractice was observed, or in case an error did occur, but did not create (health)injury for the patient. In these cases, no pay-outs are granted. Sometimes, a settlement is made between insurance company and patient. A settlement indicates that liability is not acknowledged, but a financial settlement is arranged to dismiss the claim and avoid further litigation. Motivations for a settlement may be grave injury, complex cases regarding negligence or the burden of proof, or the wish to avoid long-lasting expensive litigation process. Insurance companies will pay damages despite not admitting liability.

An *incident* is a negative, unexpected or unforeseen event that, in this study, has led to the filing of a claim.

### Statistics

The data were analysed using Excel [9].

## Results

### Subject of claims submitted

**Fig. 1:**
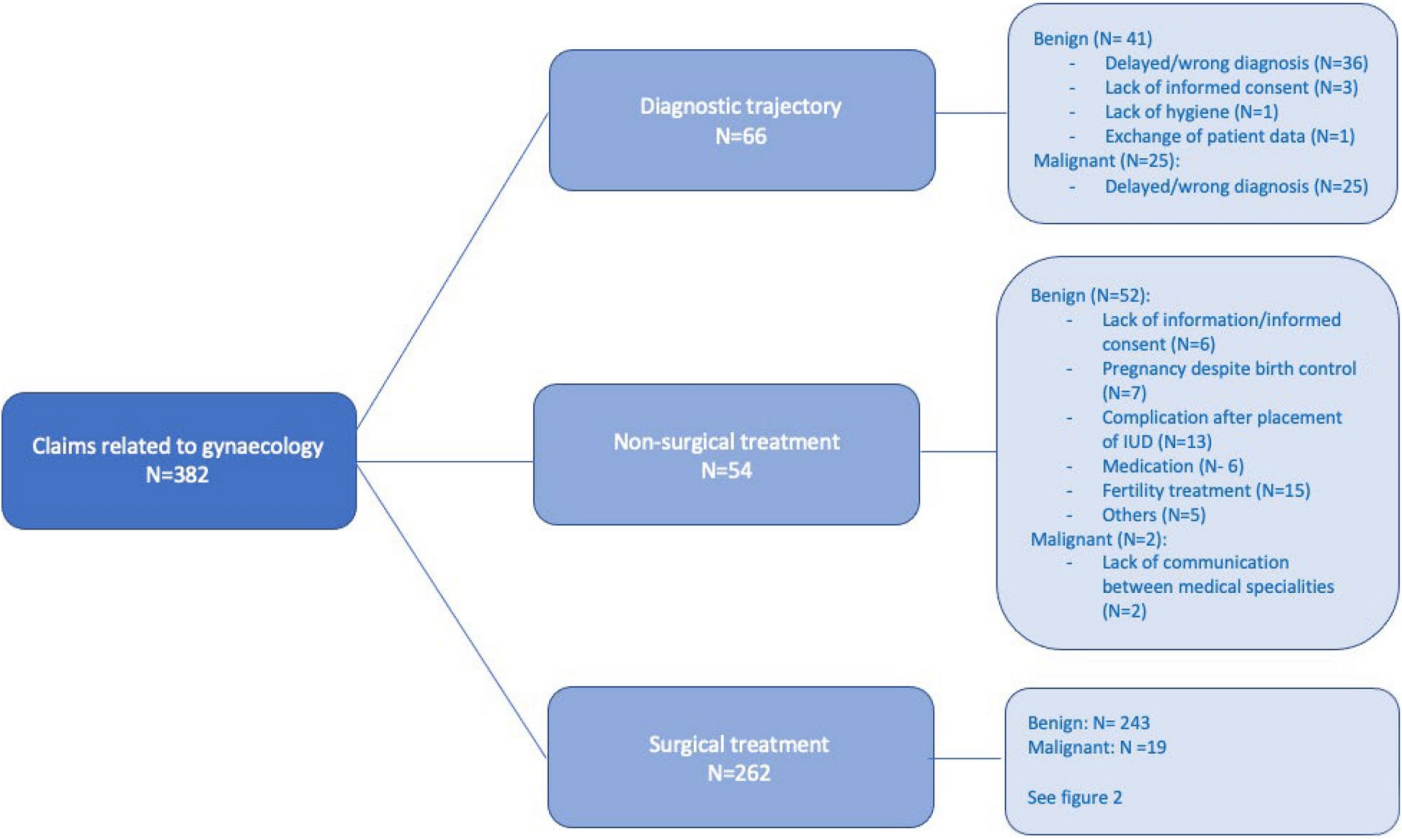
All claims filed, categorized per phase of treatment

Figure 1 shows in which healthcare phase the claimed incidents occurred. The primary claim element is leading in this figure. We divided the claims into the diagnostic and therapeutic phases. The therapeutic phase is further divided into surgical and non-surgical treatments. Per phase/group, we divided the claims based on the indication for diagnosis/treatment, knowing: benign versus malignant (cancer-related) healthcare problems.

We combined the phase in which the incident occurred with the indication for medical treatment. A total of 88.0% of all claims are related to treatments with a benign indication and only 12.0% are related to malignancies. The share of malignant treatment indications was high for claims related to diagnostic incidents (37.9%), compared to 7.3% for claims related to surgical treatment.

Overall, 17.3% of claims related to incidents in the diagnostic phase, 14.1% related to nonsurgical treatments (such as outpatient placement of an intra-uterine device (IUD) and fertility treatments), and 68.6% is related to incidents during surgical treatment. In the diagnostic phase, 37.9% of claims were related to a treatment for malignant disease, in the nonsurgical treatment phase 3.7%; and in the surgical treatment phase, 7.3%.

**Fig. 2:**
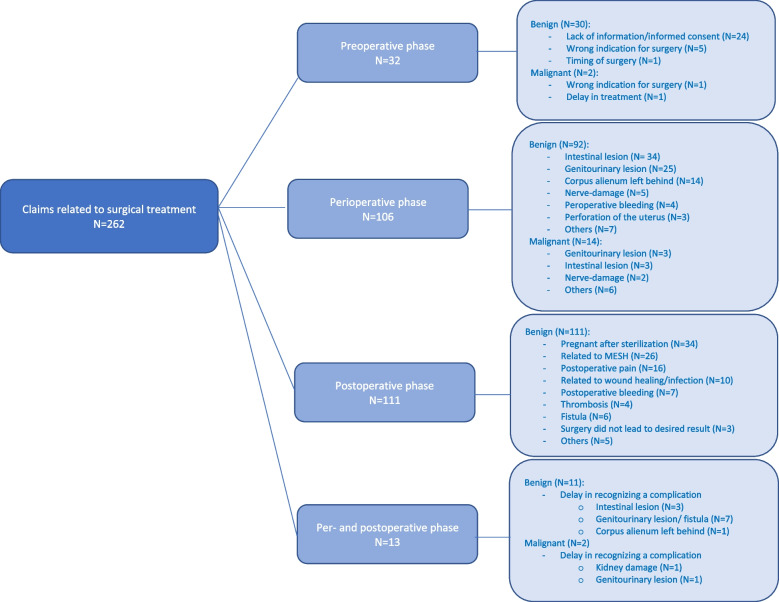
Claims related to a surgical treatment

We further categorized the surgical claims into preoperative, perioperative and postoperative incidents (Fig. 2).

Of all claims related to surgical treatment, 12.2% were related to preoperative incidents or lack of preoperative care, such as failure to provide information or lack of informed consent. In 40.5% of the cases, perioperative complications, such as intestinal lesions, genitourinary lesions and bleeding, occurred. In 42.4% of the cases, the claim focused on a (long term) incident after surgery, such as pregnancy after sterilization, complaints or concerns after placement of a MESH, postoperative pain, problems with wound healing or thrombosis. In 5.0% of the claims, the incident occurred in both the per- and postoperative phases (such as a delay in recognizing a surgical complication).

### Interval between incident and claim

As mentioned in the introduction, one possible explanation for the relatively high number of claims related to surgical specialisms and surgical treatment, may be that in the case of surgery, the occurrence of unwanted outcomes of treatment often appears sooner after a (surgical) treatment compared to diagnosis-related errors. We therefore analysed the median interval between the date of the incident and the date on which the claim was filed. We chose to analyse the median interval to minimize the effect of rare claims with very short, or excessively long intervals between incidents and claims.

In all claims related to a surgical treatment, the median interval between the incident and the filing of the claim was 498 days. In the nonsurgical treatment group, the median interval was 442 days. In the claims related to the diagnostic treatment phase, the median interval between the incident and claim was 636 days.

### Outcome of claims and costs

A total of 382 medical liability claims related to gynaecology was analysed. Liability was rejected in 187 claims (49.0%) and accepted in 86 claims (22.5%). A settlement was made in 85 claims (22.3%). In 24 claims, there was no final judgement of the insurance company (6.3%). Claims related to a malignant treatment indication are more often accepted (41.3%) compared to benign treatment-related claims (19.9%).

The total costs of all claims closed, amount to €6,632,244. The mean cost per claim was €17,362, with the highest mean costs in the accepted claims (€52,066 per accepted claim) compared to €20,922 in the claims that were settled without acknowledgement of liability and €1,836 in the claims where liability was rejected. However, the median costs per claim are markedly lower; €19,878 per accepted claims, compared to €8,624 in claims that were settled without acknowledgement of liability and €1,888 in claims where liability was rejected. For 281 claims (73.6%), total costs are less than €10,000. For 40 claims (10.5%), the costs were €10,000-€25,000; for 30 claims (7.9%) the costs were €25,000–50,000; and for 31 claims (8.1%), the costs were more than €50,000.

Of all claims related to the diagnostic phase of treatment, 31.8% were accepted. The mean costs of these accepted claims amount to €102,146. In the nonsurgical treatment phase, 20.4% of the claims were accepted. The mean costs of these accepted claims amount to €26,463. In the surgical phase, 20.6% of all claims were accepted. The mean costs of these accepted claims amount to €37,806. The highest costs per claim amounted to €764,383 and were related to a missed (oncological) diagnosis.

### Accepted claims

**Fig. 3:**
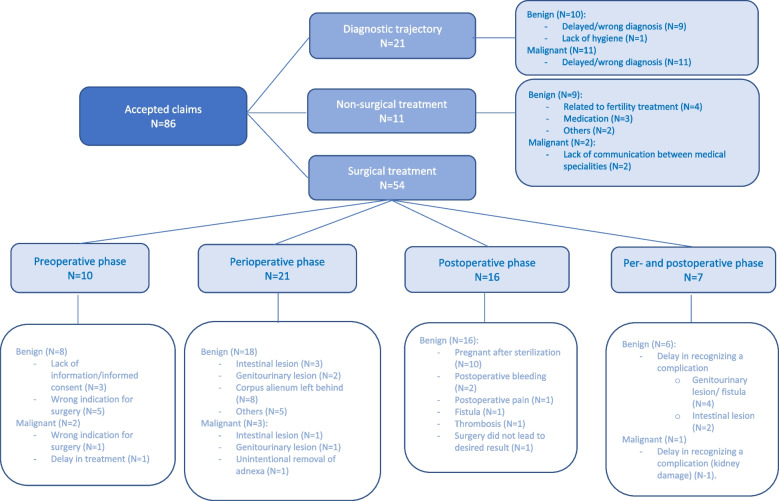
All accepted claims

In the same way that we classified all claims per phase and per indication, we classified the accepted claims (Fig. 3).

A claim may include more than one element. For example, a combination of a perioperative complication or error and a delay in recognition and treatment of this complication or error may occur. Although inadequate medical file management never occurred as the primary reason for filing a medical liability claim, an inadequate medical file had a part in 16% of all claims filed. Among the claims in which the medical file was judged to be inadequate, 30% were rejected, 26% were accepted and 43% were settled.

## Discussion

Previous studies in several countries have confirmed that medical malpractice claims are mostly related to surgical specialties compared to non-surgical and supporting medical specialties. A nationwide 15-year (2007–2021) overview of hospital-related malpractice claims in the Netherlands revealed that 64% of all claims were aimed at surgical specialties, 18% at nonsurgical (contemplative) specialties and 11% at supporting specialties. There is no correlation between this ratio and the financial turnover (as a proxy for the production numbers) of these medical specialties [3]. At the same time, a Dutch analysis of the nature, severity and extent of healthcare-related damage in hospitals in 2019 revealed that 21% of all healthcare-related damage is caused by surgical treatments, 32% is related to medication errors, and 6% of healthcare-related damage is caused in the diagnostic phase. Incidents in the diagnostic phase were more likely to be avoidable than surgical incidents (83% vs. 39%) [10].

It is remarkable that there is a relatively low percentage of healthcare-related damage caused in the perioperative phase and low amounts of avoidable surgical incidents [5], and, on the other hand, a high number and costs of claims related to surgical specialties [11]. This indicates that surgical specialties are at greater risk for malpractice claims than nonsurgical specialties are. There might be surgical-specific characteristics that increases the risk of liability claims.

As mentioned in the introduction, gynaecology is one of the medical specialties with the most (expensive) malpractice claims. A better understanding of the underlying causes of errors and claims may lead to a better understanding of the differences between claims in surgical and nonsurgical specialties. In addition, a better understanding of the events and processes that lead to claims might help to prevent future incidents, help to reduce malpractice-related costs and therefore might help to improve the quality of healthcare.

### Surgical vs nonsurgical claims

This analysis of medical liability claims shows that *within* a surgical specialty, most claims (68.6%) are related to incidents in the surgical treatment phase, 17.3% in the diagnostic phase and 14.1% in a nonsurgical treatment phase.

Various hypotheses can be posed for the different amounts and costs of claims related to surgical specialties compared to nonsurgical and supporting specialties. First, in the case of a perioperative incident, there is a specific moment when an identifiable doctor is involved, who intervened to improve the patient’s health or quality of life. If the intended effect of the intervention does not occur, the perceived health damage for the patient can most often be clearly traced back to that specific intervention and the healthcare workers involved. Second, the fact that the interval between surgery and the occurrence of perioperative complications is usually short may also contribute to the willingness to file a malpractice claim. When there is an alleged error in the diagnostic/contemplative phase, which will normally establish after a longer period of time, this is different since there will be more uncertainty about how the patient’s illness or health would have developed if the correct diagnosis was made earlier.

The findings presented in paragraph 3.2 confirm this: the median intervals between incident and filing the claim were significantly lower for (surgical) treatments (median 442–498 days) compared to the claims related to diagnostics (median 636 days).

### Treatment indications (benign vs. malignant)

We found that only 12.0% of all claims were related to malignancies. Most of these claims are related to diagnostic incidents. Claims related to incidents in per- or postoperative complications in the malignant treatment group are rare. This is remarkable since surgeries for malignancies are more extensive than benign procedures. Ideally, we would correlate these claim data to numbers of patients treated for benign or malignant diseases, or the number of surgical procedures performed by the hospitals included in this analysis. Unfortunately, these data are not available. National numbers cannot easily be extrapolated or correlated to our claim data. However, with an increase of minimal invasive and medicated treatments for benign gynaecological diagnoses, we assume that a large proportion of surgical procedures will be performed for malignant indication. Malignant procedures have a higher complication risk than benign-indicated surgeries (1–2% for benign gynaecological surgery, vs. 9–14% for malignant gynaecological disease surgery) [12, 13]. That is why it is remarkable that there are relatively few claims related to (surgical) treatment of malignant diseases.

A possible explanation for the high number of claims related to benign surgery is the thought that people who undergo elective surgery are more prone to claim if the outcome of their treatment/surgery does not meet their expectations. If the indication for surgery is not life-threatening, negative side-effects or outcomes might be more difficult to overcome. People who need surgery for cancer, may be willing to accept complications in order to recover and survive. Another possible explanation might be that, because the malignancy-related surgeries are known for their greater complication risk, patients are better informed about these risks in the perioperative phase, leading to better expectation management. Figure 2 affirms this, showing that in the benign group, 80% of claims regarding the preoperative phase were related to informed consent, versus 0% in the malignant group. Finally, severe complications are rare in benign gynaecological surgery. This might lead to (doctors’) delay in the diagnosis and treatment of complications. In particular, bowel injuries associated with gynaecologic laparoscopy have a delayed diagnosis in 41% of cases [14]. Previous findings from Barbieri et al. endorse this, stating that general gynaecologists are more prone to liability claims than are subspecialists (28–32% vs. 4–12%) [15], which could be due to information and expectation management. A delay in the recognition of a complication might lead to a more severe outcome and therefore increase the risk of a malpractice claim after a complication.

Claims related to a malignant treatment indication were more often accepted (41.3%) than claims related to a benign treatment indication (19.9%). This indicates that if a patient (or relative) with a malignant diagnosis submits a malpractice claim, there is a greater chance of a culpable error, leading to an accepted claim.

### Outcomes of claims

Of all the claims, 22.5% are accepted. Most claims are rejected, or a settlement is made. The number of accepted claims was 31.8% in the diagnostic trajectory, mostly due to an incorrect, missed or delayed (oncological) diagnosis. In the nonsurgical treatment phase, 20.4% of claims were accepted, mostly due to a lack of informed consent or communication. In the surgical treatment phase, 20.6% of the claims were accepted. The costs of accepted claims related to an incident in the diagnostic phase are remarkably greater than those of (non)surgical claims (paragraph 3.3).

A perioperative complication rarely leads to an accepted claim since perioperative complications may occur without a culpable error. This knowledge is very important for healthcare professionals. Claims related to a combination of a complication or an error, negligence in recognizing a complication, defective file management and/or a lack of informed consent are more often considered culpable and are therefore accepted. Informed consent contributes to the expectation management of patients. Insufficient informed consent may therefore increase the risk of malpractice claims. Defective file management compromises legal defence and leads to delays in the judgement of malpractice claims. In 16% of the analysed claims, the medical file is judged to be inadequate, of which 70% is accepted or a settlement is made.

## Conclusion

Gynaecology is one of the medical specialties with the most (expensive) malpractice claims. Most of the gynaecology-related claims are related to perioperative incidents, while claims related to the diagnostic phase of treatment have a greater chance of acceptance and receiving compensation payments.

The in-depth analysis of claims related to gynaecology strongly suggest that the indication for treatment (benign vs. malignant) influences the likelihood of liability. For future research, we suggest that claim-data would be correlated to the number of patients diagnosed and treated, the number of surgical procedures and the number of complications and adverse events registered. Although people under treatment for a malignant disease seem less likely to file a claim, claims related to malignancies (especially a delay or missed diagnosis) are more often accepted and have the highest indemnity payments. Besides the indication for treatment, deficient expectation management (a lack of informed consent) may contribute to dissatisfaction and increase the risk of malpractice claims. Finally, an inadequate medical file compromises legal defence and influences the judgement and settlement of malpractice claims.

The difference between the risk of claims for benign and malignant treatment observed in these gynaecological cases, might also influence the claims for all medical specialties. This might be an explanation for the high number of claims regarding general surgery and orthopaedic surgery, which are both specialties with high amounts of elective and benign surgical treatments.

## Data Availability

Data is provided within the manuscript. The datasets used and analysed during the current study are available from the corresponding author on reasonable request.
